# Shared Decision-Making Training for Home Care Teams to Engage Frail Older Adults and Caregivers in Housing Decisions: Stepped-Wedge Cluster Randomized Trial

**DOI:** 10.2196/39386

**Published:** 2022-09-20

**Authors:** Évèhouénou Lionel Adisso, Monica Taljaard, Dawn Stacey, Nathalie Brière, Hervé Tchala Vignon Zomahoun, Pierre Jacob Durand, Louis-Paul Rivest, France Légaré

**Affiliations:** 1 Tier 1 Canada Research Chair in Shared Decision Making and Knowledge Translation Québec, QC Canada; 2 VITAM – Centre de recherche en santé durable Quebec, QC Canada; 3 Department of Social and Preventive Medicine Faculty of Medicine Université Laval Quebec, QC Canada; 4 Clinical Epidemiology Program Ottawa Hospital Research Institute Ottawa, ON Canada; 5 School of Epidemiology and Public Health University of Ottawa Ottawa, ON Canada; 6 School of Nursing University of Ottawa Ottawa, ON Canada; 7 Direction des services multidisciplinaires Centre intégré universitaire de santé et de services sociaux de la Capitale-Nationale Quebec, QC Canada; 8 Knowledge Translation and Implementation Component of the Quebec Strategy for Patient-Oriented Research - Support for People and Patient-Oriented Research and Trials Unit Health and Social Services Systems Quebec, QC Canada; 9 School of Physical and Occupational Therapy Faculty of Medicine McGill University Montreal, QC Canada; 10 Department of Mathematics and Statistics Université Laval Quebec, QC Canada; 11 Canada Research Chair in Statistical Sampling and Data Analysis Laval University Quebec, QC Canada; 12 Department of Family Medicine and Emergency Medicine Faculty of Medicine Université Laval Québec, QC Canada

**Keywords:** shared decision-making, home care, nursing homes, patient engagement

## Abstract

**Background:**

Frail older adults and caregivers need support from their home care teams in making difficult housing decisions, such as whether to remain at home, with or without assistance, or move into residential care. However, home care teams are often understaffed and busy, and shared decision-making training is costly. Nevertheless, overall awareness of shared decision-making is increasing. We hypothesized that distributing a decision aid could be sufficient for providing decision support without the addition of shared decision-making training for home care teams.

**Objective:**

We evaluated the effectiveness of adding web-based training and workshops for care teams in interprofessional shared decision-making to passive dissemination of a decision guide on the proportion of frail older adults or caregivers of cognitively-impaired frail older adults reporting active roles in housing decision-making.

**Methods:**

We conducted a stepped-wedge cluster randomized trial with home care teams in 9 health centers in Quebec, Canada. Participants were frail older adults or caregivers of cognitively impaired frail older adults facing housing decisions and receiving care from the home care team at one of the participating health centers. The intervention consisted of a 1.5-hour web-based tutorial for the home care teams plus a 3.5-hour interactive workshop in interprofessional shared decision-making using a decision guide that was designed to support frail older adults and caregivers in making housing decisions. The control was passive dissemination of the decision guide. The primary outcome was an active role in decision-making among frail older adults and caregivers, measured using the Control Preferences Scale. Secondary outcomes included decisional conflict and perceptions of how much care teams involved frail older adults and caregivers in decision-making. We performed an intention-to-treat analysis.

**Results:**

A total of 311 frail older adults were included in the analysis, including 208 (66.9%) women, with a mean age of 81.2 (SD 7.5) years. Among 339 caregivers of cognitively-impaired frail older adults, 239 (70.5%) were female and their mean age was 66.4 (SD 11.7) years. The intervention increased the proportion of frail older adults reporting an active role in decision-making by 3.3% (95% CI –5.8% to 12.4%, *P*=.47) and the proportion of caregivers of cognitively-impaired frail older adults by 6.1% (95% CI -11.2% to 23.4%, *P*=.49). There was no significant impact on the secondary outcomes. However, the mean score for the frail older adults’ perception of how much health professionals involved them in decision-making increased by 5.4 (95% CI −0.6 to 11.4, *P*=.07) and the proportion of caregivers who reported decisional conflict decreased by 7.5% (95% CI −16.5% to 1.6%, *P*=.10).

**Conclusions:**

Although it slightly reduced decisional conflict for caregivers, shared decision-making training did not equip home care teams significantly better than provision of a decision aid for involving frail older adults and their caregivers in decision-making.

**Trial Registration:**

ClinicalTrials.gov NCT02592525; https://clinicaltrials.gov/show/NCT02592525

## Introduction

Aging is associated with a higher risk of developing disabilities that can lead to loss of autonomy [1,2]. When frail older adults start to lose autonomy, one of the most difficult decisions they face is whether to remain at home, with or without assistance, or move into residential care [3]. When these older adults have cognitive impairment, caregivers may have to make the decision instead, often with little support [4]. Making this difficult decision [5] can lead to stress, decisional conflict, and regret [6].

Shared decision-making (SDM) is a process whereby health professional, patients, and their caregivers work together to make health care choices based on the best evidence and what matters most to patients [7]. SDM tools, such as decision guides, are associated with better decision quality and decision-making processes without damaging patient or health system outcomes [8]. Decision guides can increase the involvement of frail older adults and caregivers in decisions about their care while improving agreement between them and their home care teams [9].

In previous work, an interprofessional SDM (IP-SDM) training program for home care teams with a decision guide increased by 12% (compared to usual care) the proportion of caregivers who reported being active in making housing decisions for frail older adults with cognitive impairment [10]. However, other studies have shown that educational interventions may make little difference to the actual practice of SDM with older adults with cognitive impairment and their surrogate decision makers [11]. In addition, given that home care teams are already very busy, and overall awareness of SDM is increasing [12], passive dissemination of decision guides alone could be enough to increase patient engagement in decision-making [13]. However, the effectiveness of decision guides alone, compared to their use as part of a multifaceted intervention, is unknown.

The aim of this study was to evaluate the effect of adding a blended web-based and in-person training program in IP-SDM for home care teams to passive dissemination of a decision guide on the proportion of frail older adults or caregivers reporting an active role in making housing decisions, compared with passive dissemination of the decision guide alone. We hypothesized that the addition of a training program in IP-SDM to the passive dissemination of a decision guide would increase the proportion of frail older adults or caregivers reporting an active role in the decision-making process.

## Methods

### Ethics Approval

We reported this trial following the extension of Consolidated Standards of Reporting Trials (CONSORT) for stepped-wedge cluster randomized trials [14]. The trial was registered at ClinicalTrials.gov (NCT02592525) and the protocol was published [15]. Ethics committee review approval has been obtained from the Multicenter Ethics Committee of Centre intégré de santé et de services sociaux de Laval (2015-2016/01-01-E).

### Study Design

**Figure 1:**
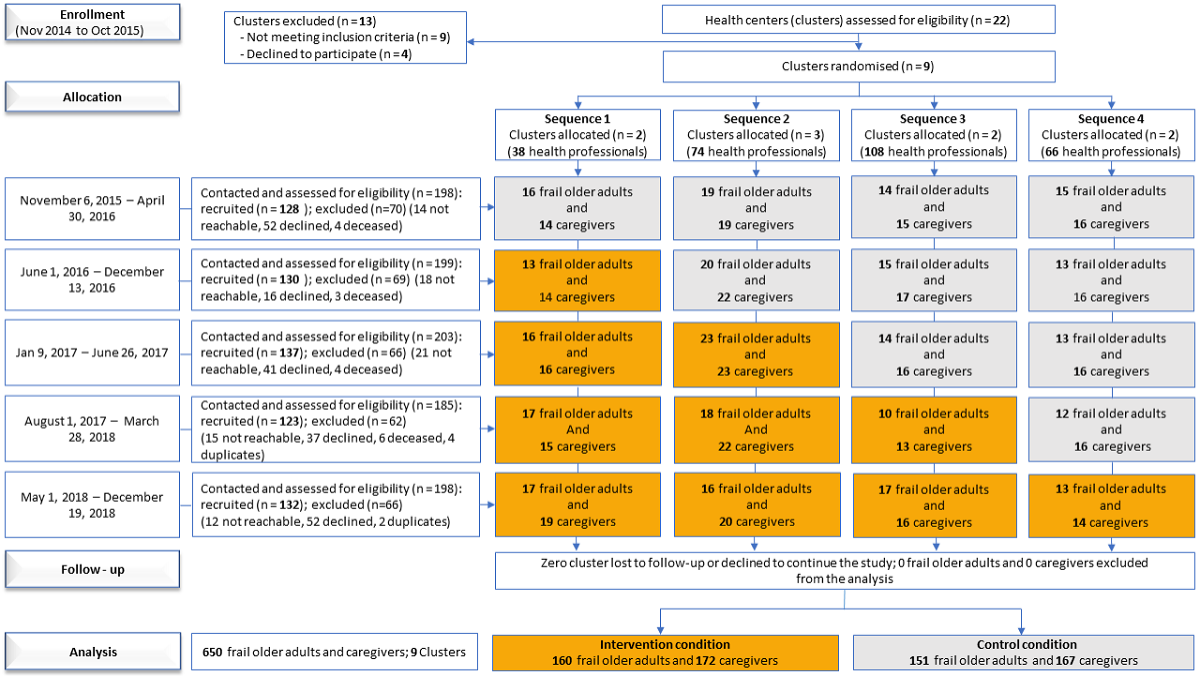
Flow chart of trial by allocated sequence and period_updated.

We conducted a cross-sectional, stepped-wedge cluster randomized trial (the Inter Professional Shared Decision-Making-Stepped Wedge Study) from November 2014 to December 2018 with home care teams at health centers in Quebec, Canada. We chose cluster randomization because the intervention was delivered at the health-center level, precluding individual randomization. A stepped-wedge design was chosen to facilitate recruitment, as all health centers would ultimately receive the intervention [16]. This design also offers more statistical power than a traditional parallel cluster study when there are large cluster-level effects (or intracluster correlations) [17]. Health centers were randomly allocated to 1 of 4 intervention start times (sequences), with 5 data collection periods (Figure 1).

### Participants and Eligibility

Study participants were frail older adults with loss of autonomy and caregivers of frail older adults with cognitive impairment who were recruited through the home care teams at the health centers. Home care teams were eligible if they (1) were involved in caring for frail older adults, (2) practiced in one of the health centers participating in the trial, and (3) were interprofessional (ie, involved more than 2 health professionals from different professions). Frail older adults were eligible if they (1) were aged ≥65 years; (2) were receiving care from one of the home care teams; (3) had made a decision about staying home or moving during the recruitment period; (4) were able to read, understand, and write French or English; and (5) were able to give informed consent. When frail older adults were cognitively impaired, their informal caregiver became the eligible participant. Caregivers were defined in this study as close relatives or friends and were eligible if they (1) were caring for a cognitively impaired older adult who was otherwise eligible; (2) were able to read, understand, and write French or English; and (3) provided informed consent to participate in the study. Frail older adults with cognitive impairment had been clinically evaluated by a health professional as no longer able to make decisions on their own.

### Randomization

Health centers (clusters) were randomized to 1 of 4 sequences. Once participating home care teams had been identified, an independent biostatistician at the Ottawa Hospital Research Institute’s Methods Centre performed randomization using computer-generated numbers. Given the nature of the intervention, the investigators, project coordinator, and research assistants (RAs) collecting the data were not blinded. However, the allocation list was concealed from the research team for as long as possible and the RAs were asked not to discuss this information with any frail older adult or caregiver and not to refer to the intervention. Frail older adults and caregivers were blinded to the intervention.

### Control

Before baseline data collection, we asked managers at all the enrolled health centers to distribute (ie, perform passive dissemination of) a decision guide for home care teams supporting frail older adults or caregivers in making housing decisions [4]. Dissemination of the decision guide was passive in the sense that although it was distributed in the health centers, we did not train the teams in how to use it. The decision guide, adapted from an online family decision support tool designed for the context of the home, had French and English versions [4,18]. It has the potential to help health professionals discuss with frail older adults or caregivers of cognitively impaired frail older adults the decision about the location of care [4,9,13].

### Intervention

The intervention consisted of (1) a 1.5-hour web-based tutorial, based on the Ottawa Decision Support Tutorial, [19] that was completed individually by the health professionals in the participating home care teams at the cluster level, followed by (2) a 3.5-hour live interactive workshop. The web-based tutorial ensured that all participants arrived at the workshop with a similar knowledge of SDM concepts. The workshop included a lecture reviewing SDM concepts (especially the IP-SDM approach); a video demonstrating the approach in a home care team with a frail older adult making a housing decision [20]; training in using the decision guide [4]; and role play using the decision guide with feedback from facilitators [15,20]. The workshop, based on adult education principles [21], included decision-making about housing decisions with frail older adults, communication techniques, and, for frail older adults with cognitive impairment, strategies for fostering their participation or that of their caregivers in decision-making. All workshops were held at the health centers, had the same content, same materials, and same trainers, and were held as a single session [15]. All home care teams received the intervention at various time points. The decision guide distributed before the intervention was still available in sufficient quantities afterwards [15]. The digital format of the initial tutorial and the video were convenient and easily scalable to our 9 intervention sites and ensured that base elements of the training were standardized and identical. This is helpful in stepped-wedge trials, where control and intervention conditions are experienced at different times, there is implementation lag, and individuals are exposed to the intervention in different ways and locations. It also reduced time expenditure and costs, in contrast to in-person training, which had to be repeated at each crossover point [22]. However, our intervention overcame the disadvantages of web-based learning (mainly isolation) [23-25] with the in-person part of the training, which provided role play, feedback, and discussion opportunities for applying knowledge to skills and behavior [26].

### Outcomes and Measurement

The primary outcome was the frail older adults’ or caregivers’ perception of the role they assumed in decision-making, as measured using a modified version of the Control Preferences Scale [27], a single question with five response categories: (1) “I made the decision,” (2) “I made the decision after seriously considering the health care professionals’ opinions,” (3) “the health care professionals and I shared the responsibility for the decision making,” (4) “the health care professionals made the decision after seriously considering my opinion,” and (5) “the health care professionals made the decision.” For sample size calculation and analysis, we dichotomized the primary outcome by collapsing categories 1, 2, and 3 into an “active” role and 4 and 5 into a “passive” role in decision-making.

Secondary outcomes assessed in frail older adults and caregivers were (1) their preferred option about whether the cognitively impaired older adult should stay at home or move to another location, and the actual decision made; (2) decisional conflict, assessed with the 16-item Decisional Conflict Scale [28,29]; (3) decision regret, assessed with the 5-item Decision Regret Scale [30]; and (4) perception of the extent to which health professionals involved them in decision-making, assessed with the Dyadic-OPTION scale, a 12-item instrument evaluating SDM behaviors during decision-making [31,32]. Other secondary outcomes included health-related quality of life, assessed only in the frail older adults with the 36 items of the Nottingham Health Profile [33-35], and burden of care, assessed only in the caregivers with the Zarit Burden Inventory scale [36-38].

### Data Collection

Home care teams made lists of potentially eligible frail older patients. Trained RAs assigned to each health center contacted these patients or caregivers of frail older adults with cognitive impairment and asked if they wished to participate. The RAs then met all interested participants at their home or a place of their choice to complete informed consent and proceed with data collection. Data collection took place from November 2015 to December 2018. Due to practical constraints, some health centers started the intervention earlier or later than planned. The collected data included outcomes; the relationship between caregivers and frail older adults (when appropriate); and sociodemographic characteristics, including age, sex, and education, which were variables identified as predictors of our primary outcome, that is, that younger, female, well-educated (secondary school level or higher) people are more likely to take an active role in decisions about their health [27,39-41].

### Sample Size

The sample size calculation was informed by preliminary data from another study [42]. We used the method developed by Hussey and Hughes [43] for stepped-wedge designs. We assumed an average of 8 frail older adults and 8 caregivers per health center in each data collection period and a time-independent intraclass correlation (ICC) of 0.05 [44]. To detect an absolute increase of 20% [45] in the primary outcome (ie, from 70% to 90%) with 80% power using a stepped-wedge design with 4 sequences and a 2-sided test at the 5% significance level, a total of 8 clusters (with a total of 320 caregivers) were required, [46] meaning 320 frail older adults and 320 caregivers of frail older adults with cognitive impairment. To account for potential loss to follow-up of clusters we recruited one more health center than planned.

### Statistical Methods

We describe organizational settings and characteristics of the health professionals randomized to the trial and report the sociodemographics of the frail older adults and caregivers using frequencies and percentages, means and SD, or medians and IQR, as appropriate. We performed analyses with the intention-to-treat principle with the frail older adult or caregiver as the unit of analysis. The primary outcome was analyzed using a generalized linear mixed model (GLMM) with logit link. The prespecified primary analysis assumed a uniform within- and between-period correlation, adjusting for time effects (categorical) and specifying a random effect for cluster [43].

We performed secondary analyses by additionally adjusting for primary outcome predictors and for imbalanced baseline characteristics [47,48]. To explore the implications of bias due to misspecification of the correlation structure [49], we conducted analyses using 2 other correlation structures identified in the literature: nested exchangeable (specifying a random cluster effect and a random time by cluster interaction) [50,51] and exponential decay (an autoregressive between-period correlation) [52]. There are no guidelines for choosing the best-fitting covariance structure, so we used the pseudo–Akaike information criterion to select the best-fitting model and presented the results as sensitivity analyses. To estimate the absolute difference, as required by the CONSORT extension for stepped-wedge cluster randomized trials, [14] we applied GLMM using an identity link with the adaptive Gaussian–Hermite approximation to the likelihood maximum [53].

For binary secondary outcomes, we conducted similar analyses. For continuous secondary outcomes, we used a linear mixed model, and summarized the intervention effects as mean differences. We obtained within-period intraclass correlation coefficients (WpICC), between-period intraclass correlation coefficients (BpICC), and cluster autocorrelation coefficients (CAC) for each outcome analyzed. We used α=.05 as the level of significance. All analyses were conducted using SAS (version 9.4, SAS Institute).

## Results

### Participants

Recruitment took place from November 2014 to December 2018. Interprofessional home care teams from 9 health centers with 281 health professionals participated in the study. Of 481 frail older adults contacted, 311 (64.6%) were recruited. Of 502 eligible caregivers contacted, 339 (67.5%) were recruited. There was no loss to follow-up of health centers, and no frail older adults, caregivers, or health centers were excluded (Figure 1). Sociodemographics of the frail older adults and caregivers were well balanced between allocated sequences (Multimedia Appendices 1 and 2).

### Baseline Characteristics of Participants

Participating frail older adults were on average 81.2 (SD 7.5) years old; 66.9% (208/311) were female and 58.8% (183/311) had secondary education or higher. Baseline characteristics were well balanced between the intervention and control groups, except for education level (Table 1). Caregivers of frail older adults with cognitive impairment were on average 66.4 (SD 11.7) years old; 70.5% (239/339) were female and 87.3% (296/339) had secondary education or higher. Most caregivers (242/339, 71.4%) were retired or at home and 90.3% (306/339) were the child, spouse, or husband of the frail older adult. Among caregivers, baseline characteristics were well balanced between the intervention and control groups, except for age (Table 2).

**Table 1:** Baseline characteristics of frail older adults without cognitive impairment (N=311).

Characteristics			Control (n=151)		Intervention (n=160)
Age (years), mean (SD)			81.6 (7.6)		80.9 (7.4)^a^
Sex (female), n (%)			101 (66.9)		107 (66.9)
**Education level, n (%)**					
	Primary school	44 (29.2)		84 (52.5)	
	Secondary school	73 (48.3)		51 (31.9)	
	Postsecondary	34 (22.5)		25 (15.6)	
**Marital status, n (%)**					
	Married/common-law partner	45 (29.8)		58 (36.3)	
	Widowed	72 (47.7)		60 (37.5)	
	Separated/divorced	20 (13.3)		25 (15.6)	
	Single	14 (9.2)		17 (10.6)	
**Household income (CAD $)^b^, n (%)**					
	Less than 30,000	83 (55.0)		86 (53.8)	
	30,000-59,999	34 (22.5)		30 (18.8)	
	60,000 and more	4 (2.7)		7 (4.4)	
	I prefer not to answer/I do not know	30 (19.9)		37 (23.1)	

^a^n=159

^b^A currency exchange rate of CAD $1=US $0.76 is applicable.

**Table 2:** Baseline characteristics of caregivers of cognitively-impaired frail older adults (N=339).

Characteristics			Control (n=167)		Intervention (n=172)
Age (years), mean (SD)			64.2 (11.9)		68.6 (11.2)
Sex (female), n (%)			122 (73.1)		117 (68.0)
**Education level, n (%)**					
	Primary school	19 (11.4)		24 (14.0)	
	Secondary school	63 (37.7)		69 (40.1)	
	Postsecondary	85 (50.9)		79 (45.9)	
**Marital status, n (%)**					
	Married/common-law partner	129 (77.2)		132 (76.7)	
	Widowed	7 (4.2)		9 (5.2)	
	Separated/divorced	16 (9.6)		18 (10.5)	
	Single	15 (9.0)		13 (7.6)	
**Household income (CAD $)^a^, n (%)**					
	Less than 30,000	37 (22.2)		43 (25.0)	
	30,000-59,999	54 (32.3)		50 (29.1)	
	60,000 or more	51 (30.5)		46 (26.7)	
	I prefer not to answer/I do not know	25 (15.0)		33 (19.2)	
**Caregivers’ employment status, n (%)**					
	Retired	94 (56.3)		114 (66.3)	
	Employed	56 (33.5)		39 (22.7)	
	At home (eg, unemployed/job seeker)	17 (10.2)		17 (9.9)	
	Missing	0 (0.0)		2 (1.1)	
**Caregivers’ relationship to frail older adult, n (%)**					
	Child	94 (56.3)		75 (43.6)	
	Wife/husband or common-law partner	59 (35.3)		78 (45.3)	
	Other (eg, family member or friend)	14 (8.4)		19 (11.1)	

^a^A currency exchange rate of CAD $1=US $0.76 is applicable.

### Primary Outcomes

At baseline (period 1), when no health center had yet received the intervention, but they had been exposed to passive dissemination of the decision guide (ie, the control condition), 92% (59/64) of frail older adults and 83% (53/64) of caregivers of frail older adults with cognitive impairment already reported an active role in decision-making (Multimedia Appendices 3 and 4). In all, 92.1% (139/151) of frail older adults recruited under the control condition reported an active role in decision-making versus 94.3% (149/160) of frail older adults recruited under the intervention condition, for an absolute increase of 3.3% (95% CI –5.8% to 12.4%, *P*=.47) after accounting for the secular trend (Table 3). Similarly, 77.8% (130/167) of caregivers recruited under the control condition reported an active role in decision-making versus 80.8% (139/172) under the intervention condition, for an absolute increase of 6.1% (95% CI –11.8% to 23.4%, *P*=.49) (Table 4) after accounting for the secular trend. The ICC (WpICC) and the CAC were, respectively, 0.051 and 0.627 in the frail older adults and 0.045 and 0.493 in the caregivers of frail older adults with cognitive impairment (Multimedia Appendices 5 and 6).

**Table 3:** Effect of the intervention on primary and secondary outcomes for frail older adults without cognitive impairment.

Outcomes			Values				Absolute scale effect size				Relative scale effect size		
			Control (n=151)		Intervention (n=160)		Proportion difference^a^/mean difference (95% CI)		*P* value		Odds ratio (95% CI)^b^		*P* value
**Primary outcome, n (%)**													
	Role assumed (active)^c^	139 (92.1)		149 (94.3)		3.3 (–5.8 to 12.4)		.47		1.70 (0.28 to 10.4)		.56	
**Secondary outcomes**													
	Preferred housing option (stay at home),^d^ n (%)	100 (66.7)		97 (60.6)		–9.4 (–27.0 to 8.2)		.29		0.65 (0.24 to 1.75)		.39	
	Housing decision made (stay at home),^d^ n (%)	41 (27.3)		61 (38.1)		3.3 (–14.1 to 20.7)		.71		1.16 (0.28 to 4.85)		.84	
	Decisional conflict (yes; scale ≥37.5), n (%)	28 (18.5)		20 (12.5)		–2.2 (–15.3 to 10.8)		.73		0.87 (0.20 to 3.74)		.85	
	Decisional regret (yes; scale >0), n (%)	107 (70.9)		108 (67.5)		–13.9 (–31.3 to 3.6)		.12		0.50 (0.12 to 2.11)		.34	
	Involvement in decision-making (Dyadic-OPTION),^e^ mean (SD)	65.8 (19.4)		67.9 (17.2)		5.8 (–0.5 to 12.1)^f^		.07		N/A^g^		N/A	
	Quality of life (0-100),^h^ mean (SD)	72.9 (23.8)		75.1 (22.3)		–2.1 (–10.0 to 5.9)^g^		.61		N/A		N/A	

^a^Generalized linear mixed model with logit link function including intervention as a binary variable, a fixed effect (categorical) for time, and specifying a random effect for cluster.

^b^Linear mixed model with dichotomous dependent variables to handle convergence issues and reported risk differences, which can be interpreted as a difference of proportions (dependent dichotomous variables coded 1/0) [54-56].

^c^n=149 and n=158 for the control and intervention groups, respectively.

^d^n=150 and n=159 for the control and intervention groups, respectively.

^e^Assessed on a continuous scale ranging from 0 to 100.

^f^Linear mixed model including intervention as binary variable, a fixed effect (categorical) for time, and specifying a random effect for cluster.

^g^N/A: not applicable.

^h^Assessed on a continuous scale ranging from 0 to 100.

**Table 4:** Effect of the intervention on primary and secondary outcomes for caregivers of cognitively-impaired frail older adults (primary analysis).

Outcomes			Outcome frequency					Absolute scale effect size					Relative scale effect size		
			Control (n=167)		Intervention (n=172)		Proportional difference^a^/mean difference (95% CI)			*P* value		Odds ratio (95% CI)^b^			*P* value
**Primary outcome, n (%)**															
	Role assumed (active)	130 (77.8)		139 (80.8)		6.1 (–11.2 to 23.4)			.49		1.30 (0.66 to 2.55)			.45	
**Secondary outcomes**															
	Preferred housing option (stay at home), n (%)	82 (49.1)		83 (48.3)		–2.7 (–19.4 to 14.1)			.76		0.89 (0.41 to 1.95)			.77	
	Housing decision made (stay at home), n (%)	27 (16.2)		36 (20.9)		2.6 (–10.0 to 15.2)			.69		1.10 (0.46 to 2.62)			.83	
	Decisional conflict (yes: scale ≥37.5), n (%)	23 (13.8)		19 (11.1)		–7.5 (–16.5 to 1.6)			.10		0.46 (0.19 to 1.11)			.08	
	Decisional regret (yes: scale >0), n (%)	117 (70.1)		124 (72.1)		1.7 (–15.0 to 18.3)			.84		1.03 (0.32 to 3.31)			.96	
	Involvement in decision making (Dyadic-OPTION),^c^ mean (SD)	69.3 (17.6)		69.4 (19.8)		1.2 (–5.2 to 7.6)^d^			.72		N/A^e^			N/A	
	Burden of care^f^ (0-88), mean (SD)	34.6 (17.2)		31.3 (16.5)		–1.1 (–6.2 to 4.0)^d^			.66		N/A			N/A	

^a^Generalized linear mixed model with adaptive Gaussian–Hermite approximation to the likelihood maximum using an identity link, including intervention as binary variable, a fixed effect (categorical) for time, and specifying a random effect for cluster.

^b^Generalized linear mixed model with logit link function, including intervention as binary variable, a fixed effect (categorical) for time, and specifying a random effect for cluster.

^c^Assessed on a continuous scale ranging from 0 to 100.

^d^Linear mixed model including intervention as binary variable, a fixed effect (categorical) for time, and specifying a random effect for cluster.

^e^N/A: not applicable.

^f^Assessed on continuous scale ranging from 0 to 88.

### Secondary Outcomes

The intervention had no statistically significant effect on any secondary outcomes among the frail older adults or caregivers. Frail older adults’ perception of the extent to which health professionals involved them in decision-making scored an average of 67 of 100 with a (nonsignificant) mean increase of 5.4 (95% CI –0.6 to 11.4; *P*=.07). For caregivers, there was a nonsignificant effect on decisional conflict: 13.8% (23/167) in the control group versus 11% (19/172) in the intervention group, for an absolute decrease of 7.5% (95% CI –16.5% to 1.6%, *P*=.10) (Tables 3 and 4).

## Discussion

### Principal Findings

This study evaluated the effectiveness of adding training in IP-SDM for home care teams to the passive dissemination of a decision guide on the proportion of frail older adults, or caregivers of frail older adults with cognitive impairment, who reported taking an active role in making a housing decision. In this pragmatic trial, we observed a nonsignificant increase in the proportion of participants reporting an active role in decision-making. We observed no significant effect on any secondary outcomes. However, for frail older adults, there was an absolute (nonsignificant) increase in the extent to which health professionals involved them in decision-making and an absolute (nonsignificant) decrease in decisional conflict among caregivers. These results lead us to make the following observations.

### Interpretation and Comparison With Prior Work

First, the nonsignificant increase observed in the primary outcome in both categories of participants (frail older adults and caregivers) may be explained by the fact that at baseline, the control group scored higher than expected. In our control condition, all clusters had been exposed to passive dissemination of the decision guide. In the trial that informed our sample size calculation (caregivers only), where the control group received usual care (ie, without the decision guide), fewer participants reported playing an active role at baseline and there was more room for improvement [10]. In the earlier trial, caregivers were also younger, and other studies confirm that younger people want a more active role in decision-making [57]. Both trials were pragmatic, and the loss of efficacy in a real clinical practice setting was to be expected. Interestingly, in both trials with caregivers of frail older adults with cognitive impairment, regardless of the decision-making role they assumed at baseline, an active decision-making role postintervention seemed to reach a similar threshold and go no further: in the first study, 79.6% took an active role postintervention [10], compared to 80.8% (139/172) in the current study. This suggests that among caregivers there is a natural ceiling to the expectation or desire to be active in decision-making on behalf of frail older adults. This threshold could be linked to discomfort with the role of being an active proxy decision-maker for more difficult and preference-sensitive decisions. At these times, it may be less stressful to surrender responsibility for decision-making to the clinician [58].

Second, we observed high staff turnover during the study. In a postintervention follow-up, we found that of the 281 health professionals who received the intervention, less than half remained, possibly due to a major restructuring of the Quebec health care system occurring at the time [46]. High staff turnover was identified as one of the main barriers to engaging in IP-SDM [59]. Thus, many participants were being cared for by staff who had not been exposed to the intervention, likely contributing to its ineffectiveness. Repeating the intervention with replacement staff could have remedied this [60]. Periodic reminders [61] and postintervention coaching could have increased the long-term effects of the intervention and fidelity to it [62]. Changing clinical, organizational, and policy-making environments can have major impacts on pragmatic trials such as ours.

Finally, the health professionals were under severe time constraints. Caregivers may have felt they should not take up too much time talking about their preferences and values, although this was suggested by the decision guide [63]. In addition, the home care teams may have felt that SDM as presented in the training would be too time-consuming, even though they may, in fact, have already been collaborating with patients and their caregivers in decision-making [7]. The perception of SDM as an issue related to the quantity of time needs to shift to a perception that SDM is rather an issue related to the quality of time [63]. Our results could be interpreted as showing that in this context, with overworked staff and high turnover, the decision aid was the most appropriate and practical intervention for increasing client involvement in decision-making.

A strength of this trial was that it was pragmatic, according to the pragmatic–explanatory continuum indicator summary (PRECIS-2) [64]. Pragmatic trials are more applicable to real clinical practice [65] and increase external validity [66]. Second, no health center was lost to follow-up, reducing selection bias and indicating that the study was relevant to its participants. Decision support for housing decisions was clearly already of great interest even before the COVID-19 pandemic and its catastrophic consequences for long-term care residents made housing decisions a policy priority [67]. Third, all analyses gave similar results, demonstrating their consistency (Multimedia Appendices 7, 8, 9, and 10).

### Limitations

This study had a number of limitations. First, we assumed our sample size would give us enough power to detect a 20% increase in our primary outcome, but the increase was 6.1% (not statistically significant). This lack of power may also explain why our study failed to detect a significant difference between the study groups, given the large CIs around their point estimates [10]. Second, identifying and recruiting participants after randomization may have increased the risk of selection bias, which would have caused under- or overestimation of the effect. However, the fact that characteristics were overall well balanced between groups indicates that this bias was minimal; we also adjusted for imbalanced variables to mitigate their influence on the estimate. Third, health professionals may have selected compliant participants, thereby inducing selection bias [68]. However, this limitation would have affected both the intervention and control groups. Fourth, the decision guide was distributed to all health professionals in the workshop. A question in our survey as to whether older adults and caregivers had been shown the decision guide should have provided us with a pseudofidelity variable regarding its use with patients [15], but due to a high level of missing data for this question, we could not include this as an outcome. It may be possible that there was a lack of fidelity to the implementation of the intervention. In this pragmatic trial, we were not able to be present at the consultations to assess this. A future mixed methods or qualitative study could provide this information and help us to better see the impact of the intervention. Finally, at the cluster level, the intervention may not be applicable in every setting, since home care services are organized differently from one jurisdiction to another [10]. At the individual level, however, the results of this study are generalizable to frail older adults and caregivers of frail older adults with cognitive impairment with similar characteristics facing housing decisions.

### Conclusions

Adding IP-SDM training to passive dissemination of a decision guide for home care teams was not sufficient to induce frail older adults or caregivers of cognitively-impaired frail older adults to take a more active role in housing decisions. Baseline involvement in decision-making was already high, suggesting that home care teams are already practicing a form of collaborative decision-making with their clients. When home care teams are overworked and understaffed, providing them with high-quality practical tools may be the best way to support them in involving their clients in decision-making. Further research could explore more effective dissemination of decision guides, a new SDM focus on time quality instead of time quantity, and how to adapt SDM interventions to crisis situations (eg, pandemics), when staff are absent or turnover is especially high.

## Appendix

Multimedia Appendix 1 Characteristic of frail elders by allocated sequence.

Multimedia Appendix 2 Characteristic of caregivers of cognitively-impaired frail elders by allocated sequence.

Multimedia Appendix 3 Marginal frequencies of the primary outcome by period and cluster for frail elderly without cognitive impairment.

Multimedia Appendix 4 Marginal frequencies of the primary outcome by period and cluster for caregivers of cognitively-impaired frail elderly.

Multimedia Appendix 5 Intraclass correlation coefficient (ICC) and cluster autocorrelation coefficient for primary and secondary outcomes among frail elders without cognitive impairment.

Multimedia Appendix 6 Intraclass correlation coefficient (ICC) and cluster autocorrelation coefficient for primary and secondary outcomes among caregivers of cognitively-impaired frail elders.

Multimedia Appendix 7 Effect of the intervention on primary and secondary outcomes for frail elders without cognitive impairment (secondary analyses).

Multimedia Appendix 8 Effect of the intervention on primary and secondary outcomes for caregivers of cognitively-impaired frail elders (secondary analyses).

Multimedia Appendix 9 Effect of the intervention on primary and secondary outcomes for frail elders without cognitive impairment using a model based on autoregressive between-period correlation (sensitivity analyses).

Multimedia Appendix 10 Effect of the intervention on primary and secondary outcomes for caregivers of cognitively-impaired frail elders using a model based on uniform between-period correlation (sensitivity analyses).

Multimedia Appendix 11 CONSORT eHEALTH Checklist (V 1.6.1).

## Abbreviations

BpICC: between-period intraclass correlation coefficient
CAC: cluster autocorrelation coefficient
GLMM: generalized linear mixed models
ICC: intraclass correlation
IP-SDM: interprofessional shared decision-making
RA: research assistant
SDM: shared decision-making
WpICC: within-period intraclass correlation coefficients
