# Supplementary material for: Shared Decision-Making Training for Home Care Teams to Engage Frail Older Adults and Caregivers in Housing Decisions: Stepped-Wedge Cluster Randomized Trial
Source: JMIR Aging. 2022 Sep 20;5(3):e39386. doi: 10.2196/39386 (PMC9533197; doi:10.2196/39386)
Supplement: Multimedia Appendix 1 [file aging_v5i3e39386_app1.docx]

**Multimedia Appendix 1.** Characteristic of frail elders by allocated sequence

|  | **Characteristics** | **Sequence 1**  **(n = 79)** | **Sequence 2**  **(n = 96)** | **Sequence 3**  **(n = 70)** | **Sequence 4**  **(n = 66)** |
| --- | --- | --- | --- | --- | --- |
| Age (years), mean (SD) | | 80.8 (7.92) | 80.7 (8.1) | 82.07 (6.9) | 81.6 (6.8) |
| Sex (Female), n(%) | | 49 (62.0) | 65 (67.7) | 51 (72.9) | 43 (65.2) |
| **Education, n(%)** | |  |  |  |  |
|  | Primary school | 39 (49.4) | 44 (45.8) | 17 (24.3) | 19 (28.8) |
|  | Secondary school | 17 (21.5) | 34 (35.4) | 26 (37.1) | 22 (33.3) |
|  | Post-secondary | 15 (19.0) | 14 (14.6) | 12 (17.1) | 21 (31.8) |
|  | None of the above | 8(10.1) | 4(4.2) | 15 (21.4) | 4(6.1) |
| **Marital status, n(%)** | |  |  |  |  |
|  | Married/common-law partner | 24 (75.6) | 32 (76.4) | 31 (75.3) | 16 (80.8) |
|  | Separated/Divorced | 15 (19.0) | 13 (13.5) | 8 (13.0) | 8 (10.2) |
|  | Single | 8 (10.3) | 8 (7.6) | 7 (9.1) | 5 (6.4) |
|  | Widowed | 5 (6.4) | 7 (6.6) | 2 (2.6) | 2 (2.6) |
| **Household income ($CAD), n(%)** | | |  |  |  |
|  | Less than 30,000 | 38 (48.10) | 55(57.3) | 41(58.6) | 35(53.0) |
|  | 30,000 – 59,999 | 16 (20.3) | 18 (18.8) | 16(22.9) | 14(21.2) |
|  | 60000 and more | 7(8.9) | 1(1.0) | 2(2.9) | 1(1.5) |
|  | I prefer not to answer/I do not know | 18(22.8) | 22(22.9) | 11(15.7) | 16(24.2) |

Abbreviations: SD, Standard deviation; $CAD, Canadian dollars
