# Supplementary material for: Shared Decision-Making Training for Home Care Teams to Engage Frail Older Adults and Caregivers in Housing Decisions: Stepped-Wedge Cluster Randomized Trial
Source: JMIR Aging. 2022 Sep 20;5(3):e39386. doi: 10.2196/39386 (PMC9533197; doi:10.2196/39386)
Supplement: Multimedia Appendix 2 [file aging_v5i3e39386_app2.docx]

**Multimedia Appendix 2.** Characteristic of caregivers of cognitively-impaired frail elders by allocated sequence

|  | **Characteristics** | **Sequence 1**  **(n = 78)** | **Sequence 2**  **(n = 106)** | **Sequence 3**  **(n = 77)** | **Sequence 4**  **(n = 78)** |
| --- | --- | --- | --- | --- | --- |
| Age (years), mean (SD) | | 66.6 (10.1) | 67.5 (12.6) | 65.9 (11.5) | 65.3 (12.3) |
| Sex (female), n(%) | | 56 (71.8) | 71 (67.0) | 55 (71.4) | 57 (73.1) |
| **Education, n(%)** | |  |  |  |  |
|  | Primary school | 11 (14.1) | 18 (17.0) | 4 (5.2) | 7 (9.0) |
|  | Secondary school | 34 (43.6) | 49 (46.2) | 28 (36.4) | 21 (26.9) |
|  | Post-secondary | 33 (42.3) | 39 (36.8) | 45 (58.4) | 50 (64.1) |
| **Marital status, n(%)** | |  |  |  |  |
|  | Married/common-law partner | 59 (75.6) | 81 (76.4) | 58 (75.3) | 63 (80.8) |
|  | Separated/divorced | 6 (7.7) | 10 (9.4) | 10 (13.0) | 8 (10.2) |
|  | Single | 8 (10.3) | 8 (7.6) | 7 (9.1) | 5 (6.4) |
|  | Widowed | 5 (6.4) | 7 (6.6) | 2 (2.6) | 2 (2.6) |
| **Employment status, n (%)** | |  |  |  |  |
|  | Retired | 49 (62.8) | 69 (65.1) | 49 (63.6) | 41 (52.5) |
|  | Employed | 20 (25.6) | 26 (24.5) | 19 (24.7) | 30 (38.5) |
|  | At home | 7 (9.0) | 8 (7.6) | 6 (7.8) | 3 (3.8) |
|  | Other (e.g., Unemployed/Job seeker) | 2 (2.6) | 3 (2.8) | 3 (3.9) | 2 (2.6) |
|  | Missing | 0 | 0 | 0 | 2 (2.6) |
| **Household income ($CAD), n(%)** | | |  |  |  |
|  | Less than 30,000 | 23 (29.5) | 30 (28.3) | 14 (18.2) | 13 (16.7) |
|  | 30,000 – 59,999 | 21 (26.9) | 32 (30.2) | 24 (31.1) | 27 (34.6) |
|  | 60,000 and more | 20 (25.6) | 21 (19.8) | 25 (32.5) | 31 (39.7) |
|  | I prefer not to answer/I do not know | 14 (18.0) | 23 (21.7) | 14 (18.2) | 7 (9.0) |
| **Relationship to older adult, n (%)** | |  |  |  |  |
|  | Child | 44 (56.4) | 47 (44.3) | 41 (53.2) | 37 (47.4) |
|  | Wife/husband or common-law partner | 27 (34.6) | 44 (41.5) | 33 (42.9) | 33 (42.3) |
|  | Other (e.g. Friend, other family member) | 7 (9.0) | 15 (14.2) | 3 (3.9) | 8 (10.3) |

Abbreviations: SD, Standard deviation; $CAD, Canadian dollars
