# Supplementary material for: Shared Decision-Making Training for Home Care Teams to Engage Frail Older Adults and Caregivers in Housing Decisions: Stepped-Wedge Cluster Randomized Trial
Source: JMIR Aging. 2022 Sep 20;5(3):e39386. doi: 10.2196/39386 (PMC9533197; doi:10.2196/39386)
Supplement: Multimedia Appendix 3 [file aging_v5i3e39386_app3.docx]

**Multimedia Appendix 3.** Marginal frequencies of the primary outcome by period and cluster for frail elderly without cognitive impairment

| **Sequence1** | Cluster 5 | 7/8  **(87.5%)** | 5/5  **(100%)** | 7/8  **(87.5%)** | 7/9  **(77.8%)** | 9/9  **(100%)** | 35/39  **(89.7%)** |
| --- | --- | --- | --- | --- | --- | --- | --- |
|  | Cluster 8 | 7/8  **(87.5%)** | 7/8  **(87.5%)** | 8/8  **(100%)** | 8/8  **(100%)** | 7/8  **(87.5%)** | 37/40  **(92.5%)** |
| **Sequence2** | Cluster 7 | 7/8  **(87.5%)** | 7/8  **(87.5%)** | 7/8  **(87.5%)** | 7/7  **(100%)** | 4/4  **(100%)** | 32/35  **(91.4%)** |
|  | Cluster 1 | 6/6  **(100%)** | 8/8  **(100%)** | 7/7  **(100%)** | 8/8  **(100%)** | 4/4  **(100%)** | 33/33  **(100%)** |
|  | Cluster 4 | 3/5  **(60%)** | 4/4  **(100%)** | 7/8  **(87.5%)** | 3/3  **(100%)** | 7/8  **(87.5%)** | 24/28  **(85.7%)** |
| **Sequence3** | Cluster 3 | 6/6  **(100%)** | 9/9  **(100%)** | 6/6  **(100%)** | 2/3  **(66.7%)** | 9/9  **(100%)** | 32/33  **(96.9%)** |
|  | Cluster 2 | 8/8  **(100%)** | 4/6  **(66.7%)** | 5/8  **(62.5%)** | 7/7  **(100%)** | 8/8  **(100%)** | 32/37  **(86.48%)** |
| **Sequence4** | Cluster 6 | 8/8  **(100%)** | 5/5  **(100%)** | 6/7  **(85.7%)** | 8/8  **(100%)** | 8/8  **(100%)** | 35/36  **(97.2%)** |
|  | Cluster 9 | 7/7  **(100%)** | 8/8  **(100%)** | 6/6  **(100%)** | 4/4  **(100%)** | 5/5  **(100%)** | 30/30  **(100%)** |
|  |  | **Period 1** | **Period 2** | **Period 3** | **Period 4** | **Period 5** | **Total** |
