# Supplementary material for: Shared Decision-Making Training for Home Care Teams to Engage Frail Older Adults and Caregivers in Housing Decisions: Stepped-Wedge Cluster Randomized Trial
Source: JMIR Aging. 2022 Sep 20;5(3):e39386. doi: 10.2196/39386 (PMC9533197; doi:10.2196/39386)
Supplement: Multimedia Appendix 4 [file aging_v5i3e39386_app4.docx]

**Multimedia Appendix 4.** Marginal frequencies of the primary outcome by period and cluster for caregivers of cognitively-impaired frail elderly

| **Sequence1** | Cluster 5 | 6/7  (**85.7%**) | 3/4  (**75.0%**) | 6/8  (**75.0%**) | 6/7  (**85.7%**) | 7/10  (**70.0%**) | 28/36  (**77.8%**) |
| --- | --- | --- | --- | --- | --- | --- | --- |
|  | Cluster 8 | 6/7  (**85.7%**) | 7/10  (**70.0%**) | 6/8  (**75.0%**) | 8/8  (**100%**) | 7/9  (**77.8%**) | 34/42  (**81.0%**) |
| **Sequence2** | Cluster 7 | 6/7  (**85.7%**) | 2/7  (**28.6%**) | 6/8  (**75.0%**) | 4/9  (**44.4%**) | 5/5  (**100%**) | 23/35  (**63.9%**) |
|  | Cluster 1 | 5/6  (**83.3%**) | 7/8  (**87.5%**) | 6/7  (**85.7%**) | 5/8  (**62.5%**) | 6/6  (**100%**) | 29/35  (**82.9%**) |
|  | Cluster 4 | 5/6  (**83.3%**) | 5/7  (**71.4%**) | 6/8  (**75.0%**) | 4/5  (**80.0%**) | 4/9  (**44.4%**) | 24/35  (**68.6%**) |
| **Sequence3** | Cluster 3 | 6/8  (**75.0%**) | 6/7  (**85.7%**) | 7/8  (**87.5%**) | 8/8  (**100%**) | 9/9  (**100%**) | 36/40  (**90.0%**) |
|  | Cluster 2 | 6/7  (**85.7%**) | 9/10  (**90.0%**) | 6/8  (**75.0%**) | 5/5  (**100%**) | 7/7  (**100%**) | 33/37  (**89.2%**) |
| **Sequence4** | Cluster 6 | 5/8  (**62.5%**) | 5/8  (**62.5%**) | 7/8  (**87.5%**) | 5/8  (**62.5%**) | 5/5  (**100%**) | 27/37  (**73.0%**) |
|  | Cluster 9 | 8/8  (**100%**) | 6/8  (**75.0%**) | 6/8  (**75.0%**) | 6/8  (**75.0%**) | 9/9  (**100%**) | 35/41  (**85.4%**) |

**Period 1 Period 2 Period 3 Period 4 Period 5 Total**
