# Supplementary material for: Shared Decision-Making Training for Home Care Teams to Engage Frail Older Adults and Caregivers in Housing Decisions: Stepped-Wedge Cluster Randomized Trial
Source: JMIR Aging. 2022 Sep 20;5(3):e39386. doi: 10.2196/39386 (PMC9533197; doi:10.2196/39386)
Supplement: Multimedia Appendix 6 [file aging_v5i3e39386_app6.docx]

**Multimedia Appendix 6.** Intraclass correlation coefficient (ICC) and cluster autocorrelation coefficient for primary and secondary outcomes among caregivers of cognitively-impaired frail elders

| **Outcomes** | **Within-period ICC** | **Cluster autocorrelation coefficient** |
| --- | --- | --- |
| Assumed role | 0.0450 | 0.4933 |
| Preferred option | 0.0307 | 1.0000^a^ |
| Actual decision made | 0.0014 | 0.5000 |
| Decision regret (dichotomized) | 0.0942 | 0.5520 |
| Decisional conflict (dichotomized) | 0.0038 | 1.0000^a^ |
| Involvement in decision-making (D-OPTION) | 0.0058 | 1.0000^a^ |
| Burden of care | 0.1281 | 1.0000^a^ |

^a^ analysis was based on a random intercept model only because the between-cluster variance component was estimated to be 0; ICC, Intraclass correlation coefficients estimated on the proportions scale, which were obtained by linear mixed model for Hooper and Girling model. We obtained within-period intraclass correlation coefficients (WpICC), between-period intraclass correlation coefficients (BpICC) and then cluster autocorrelation coefficients (CAC) for each outcome analyzed. The CAC was obtained using BpICC/WpICC and implies a 100*(1-r)% decay per period in the correlation between observation from the same cluster. We reported such estimates from the LMM as the ICC estimate on the proportions scale relevant for sample size calculation. We used an alpha value of .05 as the level of significance
