# Supplementary material for: Shared Decision-Making Training for Home Care Teams to Engage Frail Older Adults and Caregivers in Housing Decisions: Stepped-Wedge Cluster Randomized Trial
Source: JMIR Aging. 2022 Sep 20;5(3):e39386. doi: 10.2196/39386 (PMC9533197; doi:10.2196/39386)
Supplement: Multimedia Appendix 7 [file aging_v5i3e39386_app7.docx]

**Multimedia Appendix 7**. Effect of the intervention on primary and secondary outcomes for frail elders without cognitive impairment (secondary analyses)

|  |  | **Absolute scale effect size** | | **Relative scale effect size** | |
| --- | --- | --- | --- | --- | --- |
| **Outcomes** | | **Proportions difference** ^a^ **/ mean differences (95% CI)** | ***P-*value** | **Odds Ratio** **(95% CI)** ^b^ | ***P-*value** |
| **Primary outcome** | |  |  |  |  |
|  | Role assumed (Active) ^c^ | 2.7% (-6.8% to 12.2%) | *.58* | 1.56 (0.32 to 7.50) | *.57* |
| **Secondary outcomes** | |  |  |  |  |
|  | Preferred housing option  (stay at home) ^c^ | -8.5% (-26.7% to 9.7%) | *.36* | 0.67 (0.21 to 2.19) | *.51* |
|  | Housing decision made  (stay at home) ^c^ | 6.0% (-11.8% to 23.8%) | *.51* | 1.33 (0.29 to 6.15) | *.72* |
|  | Decisional conflict  (Yes: scale ≥37.5) | -2.2% (-15.7% to 11.4%) | *.75* | 0.88 (0.14 to 5.48) | *.89* |
|  | Decisional regret  (Yes: scale >0) | -13.7% (-31.7% to 4.4%) | *.14* | 0.51 (0.12 to 2.22) | *.37* |
|  | Involvement in decision-making  (D-OPTION) ^d^ | 5.2 (-1.1 to 11.4) ^e^ | *.10* | NA | NA |
|  | Quality of life (0-100) ^f^ | -1.7 (-9.5 to 6.1) ^e^ | *.67* | NA | NA |

^a^ Linear mixed models (LMM) with dichotomous dependent variables including intervention as binary variable, a fixed effect (categorical) for time, adjusting for sex, age, education and specifying a random effect for cluster; ^b^ Generalized linear mixed models (GLMM) with logit link function including intervention as binary variable, a fixed effect (categorical) for time, adjusting for sex, age, education and specifying a random effect for cluster; ^c^ Missing (n = 2); ^d^ D-OPTION assessed on continuous scale (range from 0 to 100); ^e^ LMM including intervention as binary variable, a fixed effect (categorical) for time adjusting for sex, age, education and specifying a random effect for cluster; ^f^ Assessed only in frail elders on continuous scale (range from 0 to 100); Abbreviations : CI, confidence interval; NA = Not applicable
