# Supplementary material for: Shared Decision-Making Training for Home Care Teams to Engage Frail Older Adults and Caregivers in Housing Decisions: Stepped-Wedge Cluster Randomized Trial
Source: JMIR Aging. 2022 Sep 20;5(3):e39386. doi: 10.2196/39386 (PMC9533197; doi:10.2196/39386)
Supplement: Multimedia Appendix 8 [file aging_v5i3e39386_app8.docx]

**Multimedia Appendix 8.** Effect of the intervention on primary and secondary outcomes for caregivers of cognitively-impaired frail elders (secondary analyses)

|  |  | **Absolute scale effect size** | | **Relative scale effect size** | |
| --- | --- | --- | --- | --- | --- |
|  | **Outcomes** | **Proportions difference ^a^/ mean differences (95% CI)** | ***P-*value** | **Odds Ratio** **(95% CI)** ^b^ | ***P-*value** |
|  | **Primary outcome** |  |  |  |  |
|  | Role assumed (Active) | 5.8% (-11.8% to 23.4%) | *.52* | 1.29 (0.60 to 2.75) | *.52* |
|  | **Secondary outcomes** |  |  |  |  |
|  | Preferred housing option  (stay at home) | -7.2% (-23.3% to 8.9%) | *.38* | 0.74 (0.33 to 1.68) | *.47* |
|  | Housing decision made  (stay at home) | -2.1% (-15.2% to 11.0%) | *.75* | 0.95 (0.37 to 2.45) | *.95* |
|  | Decisional conflict  (Yes: scale ≥37.5) | -6.9% (-17.7% to 3.9%) | *.21* | 0.56 (0.25 to 1.30) | *.18* |
|  | Decisional regret  (Yes: scale >0) | 3.8% (-13.0% to 20.7%) | *.66* | 1.04 (0.33 to 3.22) | *.95* |
|  | Involvement in decision-making  (D-OPTION) ^c^ | 2.2 (-3.7 to 8.0) ^d^ | *.10* | NA | NA |
|  | Burden of care (0-88) ^e^ | - 0.7 (-5.8 to 4.4) ^d^ | *.80* | NA | NA |

^a^ Generalized linear mixed models (GLMM) with the adaptative Gaussian–Hermite approximation to the likelihood maximum using an identity link including intervention as binary variable, a fixed effect (categorical) for time, adjusting for sex, age , education and specifying a random effect for cluster; ^b^ GLMM with logit link function including intervention as binary variable, a fixed effect (categorical) for time, adjusting for sex, age, education and specifying a random effect for cluster; ^c^ D-OPTION assessed on continuous scale (range from 0 to 100); ^d^ Linear mixed model (LMM) including intervention as binary variable, a fixed effect (categorical) for time, adjusting for sex, age, education and specifying a random effect for period; ^e^ Assessed only in caregivers of cognitively-impaired frail elders on continuous scale (range from 0 to 88); Abbreviations : CI, confidence interval; NA = Not applicable
