# Supplementary material for: Shared Decision-Making Training for Home Care Teams to Engage Frail Older Adults and Caregivers in Housing Decisions: Stepped-Wedge Cluster Randomized Trial
Source: JMIR Aging. 2022 Sep 20;5(3):e39386. doi: 10.2196/39386 (PMC9533197; doi:10.2196/39386)
Supplement: Multimedia Appendix 9 [file aging_v5i3e39386_app9.docx]

**Multimedia Appendix 9.** Effect of the intervention on primary and secondary outcomes for frail elders without cognitive impairment using a model based on autoregressive between-period correlation ^a^ (sensitivity analyses)

| **Outcomes** | | **Time adjusted Odds Ratio / Mean difference (95% CI)** | ***P-*value** | **Time and covariates adjusted**  **Odds Ratio / Mean difference (95% CI)** | ***P-*value** |
| --- | --- | --- | --- | --- | --- |
| **Primary outcome** | |  |  |  |  |
|  | Role assumed (Active) | 1.24 (0.20 to 7.84) ^b, d^ | *.82* | 1.08 (0.17 to 6.64) ^c, d^ | *.94* |
| **Secondary outcomes** | |  |  |  |  |
|  | Preferred housing option  (stay at home) | 0.65 (0.24 to 1.75) ^b, e^ | *.39* | 0.62 (0.23 to 1.69) ^c, d^ | *.34* |
|  | Housing decision made  (stay at home) | 1.34 (0.40 to 4.52) ^b, e^ | *.63* | 1.61 (0.46 to 5.61) ^c, d^ | *.44* |
|  | Decisional conflict  (Yes: scale ≥37.5) | 0.87 (0.20 to 3.75) ^b^ | *.85* | 0.88 (0.14 to 5.48) ^c^ | *.89* |
|  | Decisional regret  (Yes: scale >0) | 0.50 (0.11 to 2.25) ^b^ | *.37* | 0.50 (0.11 to 2.31) ^c^ | *.37* |
|  | Involvement in decision-making (D-OPTION) ^f^ | 5.95 (-0.91 to 12.80) ^g^ | *.09* | 5.98 (-1.22 to 13.18) ^h^ | *.10* |
|  | Quality of life (0-100) ^k^ | -2.12 (-10.0 to 5.9) ^g^ | *.61* | -1.72 (-9.51 to 6.11) ^h^ | *.67* |

^a^ Using Pseudo-AIC, data from frail elders without cognitive impairment fit more with the autoregressive between-period correlation; ^b^ Generalized linear mixed models (GLMM) with logit link function model including intervention as binary variable and a fixed effect (categorical) for time specifying a random effect for period; ^c^ GLMM with logit link function model including intervention as binary variable, a fixed effect (categorical) for time, adjusting for age, sex, education and specifying a random effect for period; ^d^ Missing (n = 2); ^e^ Missing (n = 1); ^f^ D-OPTION assessed on continuous scale (range from 0 to 100); ^g^ Linear mixed model (LMM) including intervention as binary variable, a fixed effect (categorical) for time specifying a random effect for period; ^h^ LMM including intervention as binary variable, a fixed effect (categorical) for time, adjusting for age, sex and education and specifying a random effect for period; ^k^ Assessed on continuous scale (range from 0 to 100); Abbreviations: CI, confidence interval
