# Supplementary material for: Shared Decision-Making Training for Home Care Teams to Engage Frail Older Adults and Caregivers in Housing Decisions: Stepped-Wedge Cluster Randomized Trial
Source: JMIR Aging. 2022 Sep 20;5(3):e39386. doi: 10.2196/39386 (PMC9533197; doi:10.2196/39386)
Supplement: Multimedia Appendix 10 [file aging_v5i3e39386_app10.docx]

**Multimedia Appendix 10.** Effect of the intervention on primary and secondary outcomes for caregivers of cognitively-impaired frail elders using a model based on uniform between-period correlation ^a^ (sensitivity analyses)

| **Outcomes** | | **Time adjusted Odds Ratio /Proportions difference**  **(95% CI)** | ***P*-value** | **Time and covariates adjusted**  **Odds Ratio /Proportions difference (95% CI)** | ***P*-value** |
| --- | --- | --- | --- | --- | --- |
| **Primary outcome** | |  |  |  |  |
|  | Role assumed (Active) | 1.26 (0.63 to 2.50) ^b^ | *.51* | 1.24 (0.58 to 2.62) ^c^ | *.58* |
| **Secondary outcomes** | |  |  |  |  |
|  | Preferred housing option(stay at home) | 0.89 (0.41 to 1.95) ^b^ | *.77* | 0.74 (0.33 to 1.65) ^c^ | *.45* |
|  | Housing decision made  (stay at home) | 1.11 (0.46 to 2.64) ^b^ | *.83* | 0.96 (0.37 to 2.54) ^c^ | *.94* |
|  | Decisional conflict  (Yes: scale ≥37.5) | 0.46 (0.19 to 1.11) ^b^ | *.08* | 0.56 (0.25 to 1.30) ^c^ | *.18* |
|  | Decisional regret  (Yes: scale >0) | 1.03 (0.31 to 3.45) ^b^ | *.97* | 1.03 (0.32 to 3.34) ^c^ | *.96* |
|  | Involvement in decision-making (D-OPTION) ^d^ | 2.27 (-3.48 to 8.02) ^e^ | *.44* | 2.15 (-3.69 to 7.98) ^f^ | *.47* |
|  | Burden of care (0-88) ^g^ | - 1.24 (-6.42 to 3.94) ^e^ | *.64* | -0.73(-5.87 to 4.40) ^f^ | *.78* |

^a^ Using Pseudo-AIC, data from caregivers of cognitively-impaired frail elders fit more with an uniform between-period correlation; ^b^ Generalized linear mixed models (GLMM) with logit link function including intervention as binary variable, a fixed effect (categorical) for time and specifying a random cluster effect and a random time by cluster interaction; ^c^ GLMM with logit link function including intervention as binary variable, a fixed effect (categorical) for time, adjusting for age, sex, education and specifying a random cluster effect and a random time by cluster interaction; ^d^ D-OPTION assessed on continuous scale (range from 0 to 100); ^e^ Linear mixed model (LMM) including intervention as binary variable, a fixed effect (categorical) for time specifying a random effect for cluster and an interaction cluster*period random effect  ^f^ LMM including intervention as binary variable, a fixed effect (categorical) for time, adjusting for age, sex and education and specifying a random effect for cluster and an interaction cluster*period random effect; ^g^ Assessed on continuous scale (range from 0 to 88); Abbreviations : CI, confidence interval
