# Supplementary material for: Shared Decision-Making Training for Home Care Teams to Engage Frail Older Adults and Caregivers in Housing Decisions: Stepped-Wedge Cluster Randomized Trial
Source: JMIR Aging. 2022 Sep 20;5(3):e39386. doi: 10.2196/39386 (PMC9533197; doi:10.2196/39386)
Supplement: Multimedia Appendix 11 [file aging_v5i3e39386_app11.pdf]

CONSORT-EHEALTH Checklist V1.6.2 Report	Manuscript Number	39386

(based on CONSORT-EHEALTH V1.6), available at [http://tinyurl.com/consort-ehealth-v1-6].

Date completed

8/15/2022 19:16:49

by

Évèhouénou Lionel Adisso

Does the Addition of a Web-Based Training in Shared Decision Making for Home Care Teams to Providing Decision Guides Better Engage Frail Elders and Caregivers in Housing Decisions? A Stepped-Wedge Cluster Randomized Trial

TITLE

1a-i) Identify the mode of delivery in the title

"Does the Addition of a Web-Based Training in Shared Decision Making for Home Care Teams to Providing Decision Guides Better Engage Frail Elders and Caregivers in Housing Decisions? A Stepped-Wedge Cluster Randomized Trial"

1a-ii) Non-web-based components or important co-interventions in title

We were not able to add all the component of the intervention because of the number of caracters required

1a-iii) Primary condition or target group in the title

Yes. "Study participants were frail elders with loss of autonomy and caregivers of frail elders with cognitive impairment recruited through the home care teams of the health centers"

ABSTRACT

1b-i) Key features/functionalities/components of the intervention and comparator in the METHODS section of the ABSTRACT

"The intervention consisted of a 1.5-hour web-based tutorial for home care teams plus a 3.5-hour interactive workshop in interprofessional SDM using a decision guide, designed to support frail elders and caregivers in making housing decisions. The control was passive dissemination of the decision guide."

1b-ii) Level of human involvement in the METHODS section of the ABSTRACT

Yes: "The intervention consisted of a 1.5-hour web-based tutorial for home care teams"

1b-iii) Open vs. closed, web-based (self-assessment) vs. face-to-face assessments in the METHODS section of the ABSTRACT

Yes. "Participants were recruited offline: "

1b-iv) RESULTS section in abstract must contain use data

Yes. "A total of 311 frail elders were included in the analysis: (208 [66.9%] female; mean [SD] age, 81.2[7.5] years; 183 [58.8%] secondary school level or higher) and 339 caregivers of cognitively-impaired frail elders (239 [70.5%] female; mean age, 66.4[11.7] years; 269 [87.3%] secondary school level or higher). After adjusting for clustering, time effects and prespecified covariates, the intervention increased the proportion of frail elders reporting active roles in decision-making by 3.3% (95% CI -5.8% to 12.4%; P=.47) and the proportion of caregivers of cognitively-impaired frail elders by 6.1% (95% CI -11.2% to 23.4%; P=.49). There was no significant impact on secondary outcomes."

1b-v) CONCLUSIONS/DISCUSSION in abstract for negative trials

Yes. "Although it slightly reduced decisional conflict for caregivers, the web-based training in SDM did not equip home care teams significantly better than provision of a decision aid for involving frail elders and their caregivers in decision-making."

INTRODUCTION

2a-i) Problem and the type of system/solution

Yes. "The aim of this study was to evaluate the effectiveness of adding a blended web-based and in-person training program in interprofessional SDM for home care teams to passive dissemination of a decision guide on the proportion of frail elders or caregivers reporting an active role in making housing decisions, compared with passive dissemination of the decision guide."

2a-ii) Scientific background, rationale: What is known about the (type of) system

Yes. "In previous work, an interprofessional SDM training program for home care teams with a decision guide increased by 12% the proportion of caregivers who reported being active in making housing decisions for frail elders with cognitive impairment, compared to usual care.[10] However, other studies have shown that educational interventions may make little difference to the actual practice of SDM with elders with cognitive impairment and their surrogate decision makers[11]. In addition, home care teams are already very busy and overall awareness of SDM is increasing.[12] Passively disseminating decision guides alone could thus be enough to increase patient engagement in decision making.[13] However, their effectiveness alone, compared to as part of a multifaceted intervention, is unknown."

Does your paper address CONSORT subitem 2b?

Yes. "We hypothesized that the addition of a training program in IP-SDM to the passive dissemination of a decision guide would increase the proportion of frail elders or caregivers reporting an active role in the decision-making process. "

METHODS

3a) CONSORT: Description of trial design (such as parallel, factorial) including allocation ratio

Yes. "We conducted a cross-sectional stepped-wedge cluster randomized trial (the IPSDM-SW Study) from November 2014 to December 2018 with the home care teams of health centers in Quebec, Canada."

3b) CONSORT: Important changes to methods after trial commencement (such as eligibility criteria), with reasons

Yes."Due to practical constraints, some health centers started the intervention earlier or later than planned."

3b-i) Bug fixes, Downtimes, Content Changes

No Bug fixes, Downtimes or Content Changes. We change introduced is the following one : "Due to practical constraints, some health centers started the intervention earlier or later than planned."

4a) CONSORT: Eligibility criteria for participants

Yes. " Frail elders were eligible if they: (1) were aged ≥65; (2) were receiving care from the home care teams; (3) had made a decision about staying home or moving during the recruitment periods; (4) were able to read, understand and write French or English; (5) were able to give informed consent. When frail elders were cognitively-impaired, their informal caregiver became the eligible participant. Caregivers were defined in this study as close relatives or friends and were eligible if they: (1) were caring for a cognitively-impaired elder who was otherwise eligible; (2) were able to read, understand, and write French or English; and (3) provided informed consent to participate in the study."

4a-i) Computer / Internet literacy

Yes. all the health professionals of the participating home care teams were "de facto" eligible to access to the web-tutorial. "The intervention consisted of (1) a 1.5 hour web-based tutorial, based on the Ottawa Decision Support Tutorial,[19] completed individually by the health professionals of the participating home care teams at the cluster level; followed by (2) a 3.5 hour live interactive workshop"

4a-ii) Open vs. closed, web-based vs. face-to-face assessments:

Yes. Participants were recruited offline. "Study participants, recruited offline, were frail elders with loss of autonomy and caregivers of frail elders with cognitive impairment recruited through the home care teams of the health centers"

4a-iii) Information giving during recruitment

Yes. "Home care teams made lists of potentially eligible frail older patients. Trained RAs assigned to each health center contacted these patients or caregivers of frail elders with cognitive impairment and asked if they would participate. Then RAs met all interested participants at their home or a place of their choice to complete informed consent and proceed with data collection."

4b) CONSORT: Settings and locations where the data were collected

Yes. "We conducted a cross-sectional stepped-wedge cluster randomized trial (the IPSDM-SW Study) from November 2014 to December 2018 with the home care teams of health centers in Quebec, Canada"

4b-i) Report if outcomes were (self-)assessed through online questionnaires

Yes. "Self-reported data collected were outcomes, relationship between caregivers and frail elders (when appropriate) and sociodemographic characteristics including age, sex, and education, variables identified as predictors of our primary outcome: younger, female, and well-educated people (secondary school level or higher) are more likely to take an active role in decisions about their health"

4b-ii) Report how institutional affiliations are displayed

Not aplicable. We did not display institutional affiliations to potential participants. And we pay attention to recruit similar participating health centers to be able to compare results easily. In addition the results did not vary by health center (cluster)

5) CONSORT: Describe the interventions for each group with sufficient details to allow replication, including how and when they were actually administered

5-i) Mention names, credential, affiliations of the developers, sponsors, and owners

Yes. "The web-based tutorial ensured that all participants arrived at the workshop with a similar knowledge of SDM concepts. The workshop included a lecture reviewing SDM concepts (especially the interprofessional SDM approach); a video demonstrating the approach in a home care team with a frail elder making a housing decision[20]; training in using the decision guide[4]; and role play using the decision guide with feedback from facilitators.[15, 20] The workshop, based on adult education principles,[21] included decision-making about housing decisions with frail elders, communication techniques and, for frail elders with cognitive impairment, strategies for fostering their participation or that of their caregivers in decision-making. Workshops were held in health center premises and were similarly offered (same content, same materials, same trainers) on a single occasion.[15] All home care teams received the intervention at various time points. The decision guide distributed before the intervention was still available in sufficient quantities afterwards.[15] The digital format of the initial tutorial and the video were convenient and easily scalable to our 9 intervention sites, and ensured that base elements of the training were standardized and identical. This is helpful in stepped-wedge trials, where control and intervention conditions are experienced at different times, there is implementation lag, and individuals are exposed to the intervention in different ways and locations. It also reduced time expenditure and costs, in contrast to in-person training, which had to be repeated at each crossover point. [22] However, our intervention overcame the disadvantages of web-based learning (mainly isolation) [23-25], by the in-person part of the training, which provided role play, feedback and discussion opportunities for applying knowledge to skills and behavior.[26]"		
**5-ii) Describe the history/development process**		
Yes, and we provided a reference for more details."The intervention consisted of (1) a 1.5 hour web-based tutorial, based on the Ottawa Decision Support Tutorial,[19] completed individually by the health professionals of the participating home care teams at the cluster level; followed by (2) a 3.5 hour live interactive workshop. The web-based tutorial ensured that all participants arrived at the workshop with a similar knowledge of SDM concepts"		
**5-iii) Revisions and updating**		
No revision or update was made. We had one intervention and one comparator		
**5-iv) Quality assurance methods**		
Not applicable, we did not need to		
**5-v) Ensure replicability by publishing the source code, and/or providing screenshots/screen-capture video, and/or providing flowcharts of the algorithms used**		
Yes, the web component of our intervention is available online on a site whose references we have provided to ensure reproducibility. "It was accessible by registration on the site [18] and has the potential to help health professionals discuss with frail elders of caregivers of cognitively-impaired frail elders the decision about the location of care [4, 9, 13]. " ; [18] described the website.		
**5-vi) Digital preservation**		
Yes. "Dissemination of the decision guide was passive in the sense that although distributed in the health centers, we did not train the teams in how to use it. The decision guide, adapted from the online family decision support tool to the context of the home was developed in French and English versions [4, 18]." [18] is https://decisionaid.ohri.ca/ODST/		
**5-vii) Access**		
Yes. "It was accessible by registration on the site [18] and has the potential to help health professionals discuss with frail elders of caregivers of cognitively-impaired frail elders the decision about the location of care [4, 9, 13]."		
**5-viii) Mode of delivery, features/functionalities/components of the intervention and comparator, and the theoretical framework**		
yes. "The workshop, based on adult education principles,[21] included decision-making about housing decisions with frail elders, communication techniques and, for frail elders with cognitive impairment, strategies for fostering their participation or that of their caregivers in decision-making. Workshops were held in health center premises and were similarly offered (same content, same materials, same trainers) on a single occasion.[15] All home care teams received the intervention at various time points. The decision guide distributed before the intervention was still available in sufficient quantities afterwards.[15] The digital format of the initial tutorial and the video were convenient and easily scalable to our 9 intervention sites, and ensured that base elements of the training were standardized and identical. This is helpful in stepped-wedge trials, where control and intervention conditions are experienced at different times, there is implementation lag, and individuals are exposed to the intervention in different ways and locations. It also reduced time expenditure and costs, in contrast to in-person training, which had to be repeated at each crossover point. [22] However, our intervention overcame the disadvantages of web-based learning (mainly isolation) [23-25], by the in-person part of the training, which provided role play, feedback and discussion opportunities for applying knowledge to skills and behavior.[26]"		
**5-ix) Describe use parameters**		
Not applicable. There is no intended "doses" and optimal timing for use.		
**5-x) Clarify the level of human involvement**		
Yes. intervention targeted health professionals, "The intervention consisted of (1) a 1.5 hour web-based tutorial, based on the Ottawa Decision Support Tutorial,[19] completed individually by the health professionals of the participating home care teams at the cluster level; followed by (2) a 3.5 hour live interactive workshop. "		
**5-xi) Report any prompts/reminders used**		
we did not use a specific recall and this is one of the limitations of our study. "Periodic reminders[61] and post-intervention coaching could have increased long-term effects and fidelity.[62] Changing clinical, organizational and policy-making environments can have major impacts on pragmatic trials such as ours."		
**5-xii) Describe any co-interventions (incl. training/support)**		
Not applicable. In the study we did not have any co-intervention to describe.		
**6a) CONSORT: Completely defined pre-specified primary and secondary outcome measures, including how and when they were assessed**		
yes,"The primary outcome was the frail elders' or caregivers' perception of the role they assumed in decision-making as measured using a modified version of the Control Preferences Scale,[27] a single question with five response categories: (A) I made the decision, (B) I made the decision after seriously considering the healthcare professionals' opinions, (C) the healthcare professionals and I shared the responsibility for the decision making, (D) the healthcare professionals made the decision after seriously considering my opinion, and (E) the healthcare professionals made the decision. For sample size calculation and analysis, we dichotomized the primary outcome by collapsing categories A, B and C into "active role" and D and E into "passive role" in decision-making.<br>Secondary outcomes assessed in frail elders and caregivers were (1) their preferred option about whether the cognitively older adult should stay at home or move to another location, and the actual decision made; (2) decisional conflict, assessed with the 16-item Decisional Conflict Scale[28, 29]; (3) decision regret, assessed with the 5-item Decision Regret Scale [30]; and (4) and perception of the extent to which health professionals involved them in decision-making, assessed with the D-OPTION scale, a 12-item instrument evaluating SDM behaviors during decision-making.[31, 32] Secondary outcomes for frail elders alone was health-related quality of life , assessed with the 36 – items of the Nottingham Health Profile ,[33-35] and for caregivers alone, burden of care, assessed with the Zarit Burden Inventory scale.[36-38]"		
**6a-i) Online questionnaires: describe if they were validated for online use and apply CHERRIES items to describe how the questionnaires were designed/deployed**		
Not applicable. No online questionnaire was used in the study.		
**6a-ii) Describe whether and how "use" (including intensity of use/dosage) was defined/measured/monitored**		
Not applicable. For the online tutorial, no dose was relevant: the health professional have to complete the tutorial		
**6a-iii) Describe whether, how, and when qualitative feedback from participants was obtained**		
yes. "However, our intervention overcame the disadvantages of web-based learning (mainly isolation) [23-25], by the in-person part of the training, which provided role play, feedback and discussion opportunities for applying knowledge to skills and behavior.[26]"		
**6b) CONSORT: Any changes to trial outcomes after the trial commenced, with reasons**		
Yes. "We conducted a cross-sectional stepped-wedge cluster randomized trial (the IPSDM-SW Study) from November 2014 to December 2018 with the home care teams of health centers in Quebec, Canada"		
**7a) CONSORT: How sample size was determined**		
**7a-i) Describe whether and how expected attrition was taken into account when calculating the sample size**		
Yes. "The sample size calculation was informed by preliminary data from another study.[42] We used the method developed by Hussey and Hughes for stepped-wedge designs.[43] We assumed an average of eight frail elders and eight caregivers per health center in each data collection period and a time-independent intra-class correlation (ICC) of 0.05.[44] To detect an absolute increase of 20%[45] in the primary outcome (from 70% to 90%) with 80% power using a stepped-wedge design with four sequences and a two-sided test at the 5% significance level, a total of eight clusters (with a total of 320 caregivers) was required,[46] meaning 320 frail elders and 320 caregivers of frail elders with cognitive impairment. To prevent any loss to follow-up of clusters, we recruited one more health center than planned."		
**7b) CONSORT: When applicable, explanation of any interim analyses and stopping guidelines**		
yes,"The primary outcome was the frail elders' or caregivers' perception of the role they assumed in decision-making as measured using a modified version of the Control Preferences Scale,[27] a single question with five response categories: (A) I made the decision, (B) I made the decision after seriously considering the healthcare professionals' opinions, (C) the healthcare professionals and I shared the responsibility for the decision making, (D) the healthcare professionals made the decision after seriously considering my opinion, and (E) the healthcare professionals made the decision. For sample size calculation and analysis, we dichotomized the primary outcome by collapsing categories A, B and C into "active role" and D and E into "passive role" in decision-making.<br>Secondary outcomes assessed in frail elders and caregivers were (1) their preferred option about whether the cognitively older adult should stay at home or move to another location, and the actual decision made; (2) decisional conflict, assessed with the 16-item Decisional Conflict Scale[28, 29]; (3) decision regret, assessed with the 5-item Decision Regret Scale [30]; and (4) and perception of the extent to which health professionals involved them in decision-making, assessed with the D-OPTION scale, a 12-item instrument evaluating SDM behaviors during decision-making.[31, 32] Secondary outcomes for frail elders alone was health-related quality of life , assessed with the 36 – items of the Nottingham Health Profile ,[33-35] and for caregivers alone, burden of care, assessed with the Zarit Burden Inventory scale.[36-38]"		
**8a) CONSORT: Method used to generate the random allocation sequence**		

Yes. "Health centers (clusters) were randomized to one of four sequences. Once participating home care teams had been identified, an independent biostatistician at the Ottawa Hospital Research Institute's Methods Centre performed randomization using computer-generated numbers. Given the nature of the intervention, the investigators, project coordinator and research assistants (RAs) collecting the data were not blinded. However, the allocation list was concealed from the research team for as long as possible and RAs were asked not to discuss this information with any frail elder or caregiver and not to refer to the intervention. Frail elders and caregivers were blinded to the intervention."		
**8b) CONSORT: Type of randomisation; details of any restriction (such as blocking and block size)**		
Yes. "Health centers (clusters) were randomized to one of four sequences. Once participating home care teams had been identified, an independent biostatistician at the Ottawa Hospital Research Institute's Methods Centre performed randomization using computer-generated numbers. Given the nature of the intervention, the investigators, project coordinator and research assistants (RAs) collecting the data were not blinded. However, the allocation list was concealed from the research team for as long as possible and RAs were asked not to discuss this information with any frail elder or caregiver and not to refer to the intervention. Frail elders and caregivers were blinded to the intervention."		
**9) CONSORT: Mechanism used to implement the random allocation sequence (such as sequentially numbered containers), describing any steps taken to conceal the sequence until interventions were assigned**		
Yes. "an independent biostatistician at the Ottawa Hospital Research Institute's Methods Centre performed randomization using computer-generated numbers."		
**10) CONSORT: Who generated the random allocation sequence, who enrolled participants, and who assigned participants to interventions**		
Yes. "an independent biostatistician at the Ottawa Hospital Research Institute's Methods Centre performed randomization using computer-generated numbers."		
**11a) CONSORT: Blinding - If done, who was blinded after assignment to interventions (for example, participants, care providers, those assessing outcomes) and how**		
**11a-i) Specify who was blinded, and who wasn't**		
Yes. "Given the nature of the intervention, the investigators, project coordinator and research assistants (RAs) collecting the data were not blinded. However, the allocation list was concealed from the research team for as long as possible and RAs were asked not to discuss this information with any frail elder or caregiver and not to refer to the intervention. Frail elders and caregivers were blinded to the intervention."		
**11a-ii) Discuss e.g., whether participants knew which intervention was the "intervention of interest" and which one was the "comparator"**		
Yes. Particpants ehen applicable were only aware of the intervention but they did know what was the comparator. "Home care teams made lists of potentially eligible frail older patients. Trained RAs assigned to each health center contacted these patients or caregivers of frail elders with cognitive impairment and asked if they would participate. Then RAs met all interested participants at their home or a place of their choice to complete informed consent and proceed with data collection. Data collection took place from November 2015 to December 2018. Due to practical constraints, some health centers started the intervention earlier or later than planned. Self-reported data collected were outcomes, relationship between caregivers and frail elders (when appropriate) and sociodemographic characteristics including age, sex, and education, variables identified as predictors of our primary outcome: younger, female, and well-educated people (secondary school level or higher) are more likely to take an active role in decisions about their health [27, 39-41]."		
**11b) CONSORT: If relevant, description of the similarity of interventions**		
Not relevant for the study		
**12a) CONSORT: Statistical methods used to compare groups for primary and secondary outcomes**		
Yes. "The primary outcome was analyzed using a generalized linear mixed model (GLMM) with logit link. The pre-specified primary analysis assumed a uniform within- and between-period correlation, adjusting for time effects (categorical) and specifying a random effect for cluster.[43]"		
**12a-i) Imputation techniques to deal with attrition / missing values**		
Yes. Missing value rate is very low (less than 5%) and we did not use imputation method. "Missing data rate is 98%." I addition, there was no attrition dur to the crosssectional nature of the stepped wedge. However no health center was loss to follow-up or refused to continue the study. "There was no loss to follow-up of health centers and no frail elders, caregivers or health centers were excluded".		
**12b) CONSORT: Methods for additional analyses, such as subgroup analyses and adjusted analyses**		
Yes. "We performed secondary analyses by additionally adjusting for primary outcome predictors and for imbalanced baseline characteristics.[47, 48] To explore the implications of bias due to misspecification of the correlation structure,[49] we conducted analyses using two other correlation structures identified in the literature: nested exchangeable (specifying a random cluster effect and a random time by cluster interaction)[50, 51] and exponential decay (an autoregressive between-period correlation).[52] There are no guidelines for choosing the best-fitting covariance structure, so we used the pseudo-AIC information criteria to select the best-fitting model and presented the results as sensitivity analyses."		
RESULTS		
**13a) CONSORT: For each group, the numbers of participants who were randomly assigned, received intended treatment, and were analysed for the primary outcome**		
Yes. "Of 481 frail elders contacted, 311 (64.6%) were recruited. Of 502 eligible caregivers contacted, 339 (67.5%) were recruited. "		
**13b) CONSORT: For each group, losses and exclusions after randomisation, together with reasons**		
Yes . "There was no loss to follow-up of health centers and no frail elders, caregivers or health centers were excluded (Figure 1). Sociodemographics of frail elders and caregivers were well balanced between allocated sequences."		
**13b-i) Attrition diagram**		
Yes. "Figure 1: Flowchart for the trial by allocated sequence and period"		
**14a) CONSORT: Dates defining the periods of recruitment and follow-up**		
Yes. "Recruitment took place from November 2014 to December 2018. Interprofessional home care teams from nine health centers with 281 health professionals participated in the study"		
**14a-i) Indicate if critical "secular events" fell into the study period**		
No change in the the internet resources fell into the study period.		
**14b) CONSORT: Why the trial ended or was stopped (early)**		
The stepped wedge cluster randomized trial was completed		
**15) CONSORT: A table showing baseline demographic and clinical characteristics for each group**		
Yes. "Table 2 : Baseline characteristics of participants"		
**15-i) Report demographics associated with digital divide issues**		
Yes. "Participating frail elders were on average 81.2 (SD: 7.5) years old; 66.9% were female and 58.8% had secondary education or higher. Baseline characteristics were well-balanced between intervention and control except for education level (Table 1). Caregivers of frail elders with cognitive impairment were on average 66.4 years old (SD: 11.7); 70.5% were female and 87.3% had secondary education. Most caregivers (72%) were retired or at home and 90.3% were the child, spouse, or husband of the frail elder. Among caregivers, baseline characteristics were well-balanced between intervention and control, except for age (Table 2)."		
**16a) CONSORT: For each group, number of participants (denominator) included in each analysis and whether the analysis was by original assigned groups**		
**16-i) Report multiple "denominators" and provide definitions**		
Yes. "Of 481 frail elders contacted, 311 (64.6%) were recruited. Of 502 eligible caregivers contacted, 339 (67.5%) were recruited. There was no loss to follow-up of health centers and no frail elders, caregivers or health centers were excluded (Figure 1)"		
**16-ii) Primary analysis should be intent-to-treat**		
Yes. "We performed analyses by the intention-to-treat principle with the frail elder or caregiver as the unit of analysis. The primary outcome was analyzed using a generalized linear mixed model (GLMM) with logit link. The pre-specified primary analysis assumed a uniform within- and between-period correlation, adjusting for time effects (categorical) and specifying a random effect for cluster.[43]"		
**17a) CONSORT: For each primary and secondary outcome, results for each group, and the estimated effect size and its precision (such as 95% confidence interval)**		
Yes. "). In all, 92.1% of frail elders recruited under the control condition reported an active role in decision-making versus 94.3% of frail elders recruited under the intervention condition for an absolute increase of 3.3% (95% CI, -5.8% to 12.4%; P=.47) after accounting for the secular trend (Table 3). Similarly, 77.8% caregivers recruited under the control condition reported an active role in decision-making versus 80.8% under the intervention condition for an absolute increase of 6.1% (95% CI, -11.8% to 23.4%; P=.49) "		
**17a-i) Presentation of process outcomes such as metrics of use and intensity of use**		
Not applicable in out study. Our design was crossectional and no dose effect was assessed.		
**17b) CONSORT: For binary outcomes, presentation of both absolute and relative effect sizes is recommended**		
Yes. "To estimate the absolute difference, as required by the CONSORT extension for stepped-wedge cluster randomized trials,[14] when dealing with binary outcomes, we applied GLMM using an identity link with the adaptative Gaussian–Hermite approximation to the likelihood maximum.[53] "		
**18) CONSORT: Results of any other analyses performed, including subgroup analyses and adjusted analyses, distinguishing pre-specified from exploratory**		
Yes, "We performed secondary analyses by additionally adjusting for primary outcome predictors and for imbalanced baseline characteristics.[47, 48] "		
**18-i) Subgroup analysis of comparing only users**		
**19) CONSORT: All important harms or unintended effects in each group**		
We did not perform subgroup analyses		
**19-i) Include privacy breaches, technical problems**		
Not applicable, no physical harm was identified in the study		
**19-ii) Include qualitative feedback from participants or observations from staff/researchers**		

We did not perform qualitative analysis itself. "However, our intervention overcame the disadvantages of web-based learning (mainly isolation) [23-25], by the in-person part of the training, which provided role play, feedback and discussion opportunities for applying knowledge to skills and behavior.[26] No change in the outcomes was observed."		
DISCUSSION		
**20) CONSORT: Trial limitations, addressing sources of potential bias, imprecision, multiplicity of analyses**		
**20-i) Typical limitations in ehealth trials**		
Yes, "Limitations section in the Discussion"		
**21) CONSORT: Generalisability (external validity, applicability) of the trial findings**		
**21-i) Generalizability to other populations**		
Yes. "At the individual level, however, results of this study are generalizable to frail elders and caregivers of frail elders with cognitive impairment with similar characteristics facing housing decisions."		
**21-ii) Discuss if there were elements in the RCT that would be different in a routine application setting**		
We designed a pragmatic trial."Pragmatic trials are more applicable to real clinical practice[65] and increase external validity"		
**22) CONSORT: Interpretation consistent with results, balancing benefits and harms, and considering other relevant evidence**		
**22-i) Restate study questions and summarize the answers suggested by the data, starting with primary outcomes and process outcomes (use)**		
Yes. "We observed a non-significant on the outcomes."		
**22-ii) Highlight unanswered new questions, suggest future research**		
Yes. "It may be possible that there is a lack of fidelity of implementation of the intervention. In this pragmatic trial we were not able to be present in the consultations to assess this, but a future mixed-methods or qualitative study could provide this information and help us to better see the impact of the intervention"		
Other information		
**23) CONSORT: Registration number and name of trial registry**		
"The trial was registered (NCT02592525)"		
**24) CONSORT: Where the full trial protocol can be accessed, if available**		
Yes. "the protocol was published.[15]"		
**25) CONSORT: Sources of funding and other support (such as supply of drugs), role of funders**		
Defined in the protocol: [15		
**X26-i) Comment on ethics committee approval**		
yes. "Ethics committee review approval has been obtained from the Multicenter Ethics Committee of CISSS-Laval . "		
**x26-ii) Outline informed consent procedures**		
Yes, informed consent was obtained offline. "Home care teams were eligible if they: (1) were involved in caring for frail elders; (2) practiced in one of the health centers participating in the trial; and (3) were interprofessional, i.e., involved more than two health professionals from different professions, (4) provided informed consent to participate in the study. Frail elders were eligible if they: (1) were aged ≥65; (2) were receiving care from the home care teams; (3) had made a decision about staying home or moving during the recruitment periods; (4) were able to read, understand and write French or English; (5) were able to give informed consent. When frail elders were cognitively-impaired, their informal caregiver became the eligible participant. Caregivers were defined in this study as close relatives or friends and were eligible if they: (1) were caring for a cognitively-impaired elder who was otherwise eligible; (2) were able to read, understand, and write French or English; and (3) provided informed consent to participate in the study. Frail elders with cognitive impairment had been clinically evaluated by a health professional as no longer able to make decisions on their own."		
**X26-iii) Safety and security procedures**		
Procedures were completely secures. "Home care teams made lists of potentially eligible frail older patients. Trained RAs assigned to each health center contacted these patients or caregivers of frail elders with cognitive impairment and asked if they would participate. Then RAs met all interested participants at their home or a place of their choice to complete informed consent and proceed with data collection. Data collection took place from November 2015 to December 2018. Due to practical constraints, some health centers started the intervention earlier or later than planned. Self-reported data collected were outcomes, relationship between caregivers and frail elders (when appropriate) and sociodemographic characteristics including age, sex, and education, variables identified as predictors of our primary outcome: younger, female, and well-educated people (secondary school level or higher) are more likely to take an active role in decisions about their health [27, 39-41]."		
**X27-i) State the relation of the study team towards the system being evaluated**		
Yes. "None declared conflict of interest"		
